# Effects of auricular acupressure on dysmenorrhea: A systematic review and meta-analysis of randomized controlled trials

**DOI:** 10.3389/fendo.2022.1016222

**Published:** 2023-01-05

**Authors:** Xianglu Kong, Hong Fang, Xiaoqian Li, Yanjuan Zhang, Yi Guo

**Affiliations:** ^1^ Jiande hospital of integrated traditional Chinese and Western Medicine, Hangzhou, China; ^2^ The Second Clinical Medical College, Guangzhou University of Chinese Medicine, Guangzhou, China; ^3^ The 8th Clinical Medical College, Guangzhou University of Chinese Medicine, Foshan, China; ^4^ Foshan Hospital of Traditional Chinese Medicine, Foshan, China

**Keywords:** auricular acupressure, dysmenorrhea, menstrual pain, review, meta-analysis

## Abstract

**Background:**

Auricular acupressure (AA) is widely used in treatment of dysmenorrhea, but the safety and efficacy of auricular acupressure on dysmenorrhoea are still lack of evidence-based basis.

**Objective:**

The purpose of meta-analysis was to evaluate the effects of auricular acupressure on dysmenorrhea.

**Data sources:**

A systematic search was conducted in six electronic databases, including PubMed, Embase, Cochrane Central Register of Controlled Trials (CINAHL), Weipu (CQVIP), China National Knowledge Infrastructure (CNKI), and Wanfang databases, to retrieve studies published from the inception dates to June 10, 2022.

**Study selection:**

Randomized controlled trials (RCTs) that investigated the effectiveness of AA on dysmenorrhea were identified.

**Data extraction and synthesis:**

The data extraction and quality assessment of the included studies were performed by two reviewers independently. Outcomes were abstracted to determine the effect measure by using mean differences (MD), standardized mean differences (SMD), or odds ratio (OR) from a random effects model.

**Main outcomes and measures:**

Cure rate, total effective rate, and visual analogue scale (VAS) were described as primary outcomes; Short-form Menstrual Distress Questionnaire (MDQs), symptom scores, serum nitric oxide (NO) level, and adverse events were recorded as secondary outcomes.

**Results:**

Thirty-five RCTs involving 3960 participants were included in this study. Our findings indicated that, overall, AA was associated with a significant benefit in cured rate (OR = 1.95, 95%CI: [1.34, 2.83], P=0.0004, I^2^ = 75%), total effective rate (OR = 3.58, 95%CI: [2.92, 4.39], P<0.00001, I^2^ = 67%), VAS score (MD = -1.45, 95%CI: [-1.73, -1.17], P<0.00001, I^2^ = 67%), and symptom scores compared to the control group (SMD = -0.85, 95%CI: [-1.28, -0.43], P<0.0001, I^2^ = 91%). However, no difference in serum NO (SMD = 0.77, 95%CI: [-0.39, 1.92], P = 0.19, I^2^ = 89%) and MDQs (SMD = -0.58, 95%CI: [-1.26, 0.10], P = 0.10, I^2^ = 79%) was found between the two groups. Furthermore, subgroup analysis results indicated that AA showed significant superiorities in increasing cured rate and total effective rate, and reducing VAS score and symptom scores when compared to analgesics and non-intervention. Moreover, AA presented the same superiorities when used as an adjunctive strategy to other therapy. However, these benefits were not detected in AA used alone when compared to the therapies, including Chinese herbs, acupuncture, external application of Chineseherbal medicine, moxibustion, auricular needle, and health education.

**Conclusions:**

Overall, AA, as a potential safety therapy, is effective for the management of dysmenorrhea, such as increasing cured rate, total effective rate, VAS, and symptom scores. Nevertheless, AA showed no significant improvement in serum NO and MDQs. It is furtherly found that AA used alone is superior to analgesics and non-intervention regarding cured rate, total effective rate, VAS, and symptom scores. Furthermore, the same superiorities are observed when AA serves as an adjunctive strategy to other therapy. However, AA alone has little effect on them compared to other therapies, and there is no definite conclusion on the benefits of AA compared to placebo for patients with dysmenorrhea. Rigorous RCTs with blind method and placebo control are warranted to confirm these findings.

**Systematic review registration:**

https://www.crd.york.ac.uk/prospero/, identifier CRD42022338524.

## 1 Introduction

As one of the most common gynecologic complaints, dysmenorrhea always exhibits a complex of symptoms during menstruation or before it, inducing lower abdominal pain, headache, low back pain, pelvic pain, fatigue, nausea vomiting, which seriously compromises a woman’s quality of life (1). It is estimated that 50% to 90% of adolescent girls and women of reproductive age experience dysmenorrhea, and it is a leading cause of recurrent absenteeism (2). Due to its high prevalence, dysmenorrhea has been a public health concern worldwide, which attracted increasing attentions. Nevertheless, it is often underdiagnosed, and inadequately treated, and even accepted as an inevitable symptom to menstruation by patients themselves (3). To date, there are some therapeutic methods used for prevention or treatment of perimenstrual pain, such as analgesics, hormonal contraceptives, relaxation, warmth, health education, exercise, and traditional Chinese medicine (e.g., Chinese herbal decoction, acupuncture, moxibustion, auricular acupressure and so on). Nonsteroidal anti-inflammatory drugs (NSAIDs) and hormonal contraceptives are considered as first-line therapy (1). However, they mainly exert an effect on temporary pain relief, while the long-term efficacy is always unsatisfactory (4). Furthermore, adverse reactions and unnecessary medical expenses from them can’t be neglected. Thus, it is essential to develop an effective, safe and feasible therapy to alleviate dysmenorrhea. In recent years, with continuous explorations of clinical practices, complementary and alternative medicine has come into widespread use owing to its fewer unpleasant side effects and high efficiency (5).

Auricular acupressure (AA) is a noninvasive treatment in traditional Chinese medicine technique, and it has been proved to be a valuable strategy to improve menstrual symptoms through pressing vaccaria, magnetic beads and cowherb seeds sticked on auricular acupoints corresponding to all parts of the human body (6, 7). As a typical traditional Chinese medical therapy using acupoints without needle insertion, AA stimulates acupoints by pressure from fingers or automatically by the seeds themselves (8). Particularly, in Chinese medicine theory, menstrual symptoms are viewed to be caused by either stagnant qi or Blood or the lack of blood in the body. AA can reduce tension and contraction of uterine, promote wellness, and maintain the normal bodily functions through stimulating auricular acupoints to activate and adjust the flow of qi and Blood, and subsequently reduce pain, and provide comfort. As is reported previously, it can relieve pain and neuronal excitability through facilitating the normalization of pathological hypersensitive reflex pathways connecting the ear microsystem and somatotopic brain, and regulating proinflammatory cytokines, such as IL-1b, IL-6, and TNF (9). Therefore, AA has been proposed to be applied to improve dysmenorrhea. Recently, growing numbers of studies focusing on determining the efficacy of AA on dysmenorrhea. However, until now, the safety and efficacy of AA on dysmenorrhea are still lack of evidence-based basis, hence a systematic review and meta-analysis regarding AA for dysmenorrhea is required. Consequently, we aimed to evaluate the efficacy of AA on dysmenorrhea through this meta-analysis of randomized controlled trials (RCTs) to provide evidence-based support for the management of dysmenorrhea.

## 2 Methods

This study was reported following the Preferred Reporting Items for Systematic Reviews and Meta-Analyses (PRISMA) guidelines (10). Morever, the registered protocol of this study is available in PROSPERO (registration number is CRD42022338524).

### 2.1 Search strategy

In order to identify all relevant publications, 7 electronic databases including PubMed, Embase, Cochrane Central Register of Controlled Trials (CINAHL), VIP Database for Chinese Technical Periodicals (CQVIP), China National Knowledge Infrastructure (CNKI), and Wanfang databases were comprehensively searched from inception to June 10, 2022. A keyword such as “Auricular Acupressure”, “dysmenorrhea”, “randomized”, “randomized controlled trial”, etc. were used to search in each database, and we imposed no restrictions with respect to language. Reference lists of reviews published previously and Google scholar were also checked to find additional eligible studies. The detailed search strategy was described in **Supplementary eFigure 1**.

### 2.2 Selection criteria

PICOS (patients, intervention, comparator, outcomes, and study design) framework (11) was used to formulate inclusion and exclusion criteria in this study.

#### 2.2.1 Patients

Inclusion criteria:

- Subjects with primary and secondary dysmenorrhea.
- Exclusion criteria:
- Participants with gynecological tumor.
- Participants with a history of gynecological surgery.

#### 2.2.2 Interventions

Inclusion criteria:

AA should be applied in the experimental group in include study.

Exclusion criteria:

- Acupuncture,
- Auricular needle;
- Electrical acupoints stimulation.

#### 2.2.3 Comparators

AA versus (VS) basic or conventional treatment; AA + basic or conventional treatment VS basic or conventional treatment used alone; AA VS non-intervention; AA VS placebo or sham acupressure.

#### 2.2.4 Outcomes

The primary outcomes were listed as follow:

- Cure rate;
- Total effective rate;
- Pain intensity evaluated by using visual analogue scale (VAS).
- Secondary outcomes
- Short-form Menstrual Distress Questionnaire (MDQs) (12);
- Symptom scores;
- Serum nitric oxide (NO) level;
- Adverse event.

#### 2.2.5 Study design

Inclusion criteria:

- Clinical RCT;
- Published in a peer-reviewed journal;
- Exclusion criteria:
- Repeated publications;
- Comments, protocol, conference abstracts, meta-analysis, or reviews;
- The data were incomplete;
- Full-text unavailable literatures.

### 2.3 Study screening and data extraction

All the search results were imported to EndNote X8 (Bld 10063), a reference management software, to remove duplicate articles. Two independent researchers (XL Kong and H Fang) screened the retrieved literatures by reading the titles, abstracts, and full text, and then identify the included studies in accordance with the aforementioned criteria. Next, the two reviewers independently extracted relevant data from the included studies, including the author, publication year, sample size, auricular acupoints, intervention parameters, study design, and outcomes. When screening and extracting data, we resolved discrepancies through discussion until a consensus was reached.

### 2.4 Quality assessment

The Cochrane Collaboration’s Risk of Bias tool was used to assess the quality of the included studies by the two independently reviewers from seven domains including random sequence generation, allocation concealment, blinding of participants and personnel, blinding of outcome assessment, incomplete outcome data, selective reporting, and other bias (13). The risk of each item is categorized as high, unclear and low.

### 2.5 Statistical analysis

All the data analysis were performed by using review manager (version 5.3, the Nordic Cochrane Centre, Copenhagen, Denmark) and Stata (version 13.0, the StataCorp LP, USA). For continuous outcomes, mean difference (MD) standardized mean difference (SMDs) with 95% confidence intervals (95% CI) were reported as the effect size. Meanwhile, dichotomous data were presented with as risk ratio (RR) with 95% CI. Moreover, the statistical heterogeneity was tested by using the Cochran Q-test and I^2^. If I^2^ statistic smaller than 50%, we considered the heterogeneous was acceptable (14) and a fixed effects model was adopted. Otherwise, the data was analyzed by using a random effects model (I^2^ > 50%). Subgroup analyses were performed based on different comparators. In addition, Begg’s test and Egger’s test was conducted to estimate publication bias. The significant difference level was set at P < 0.05.

## 3 Results

### 3.1 Study selection

**Figure 1:**
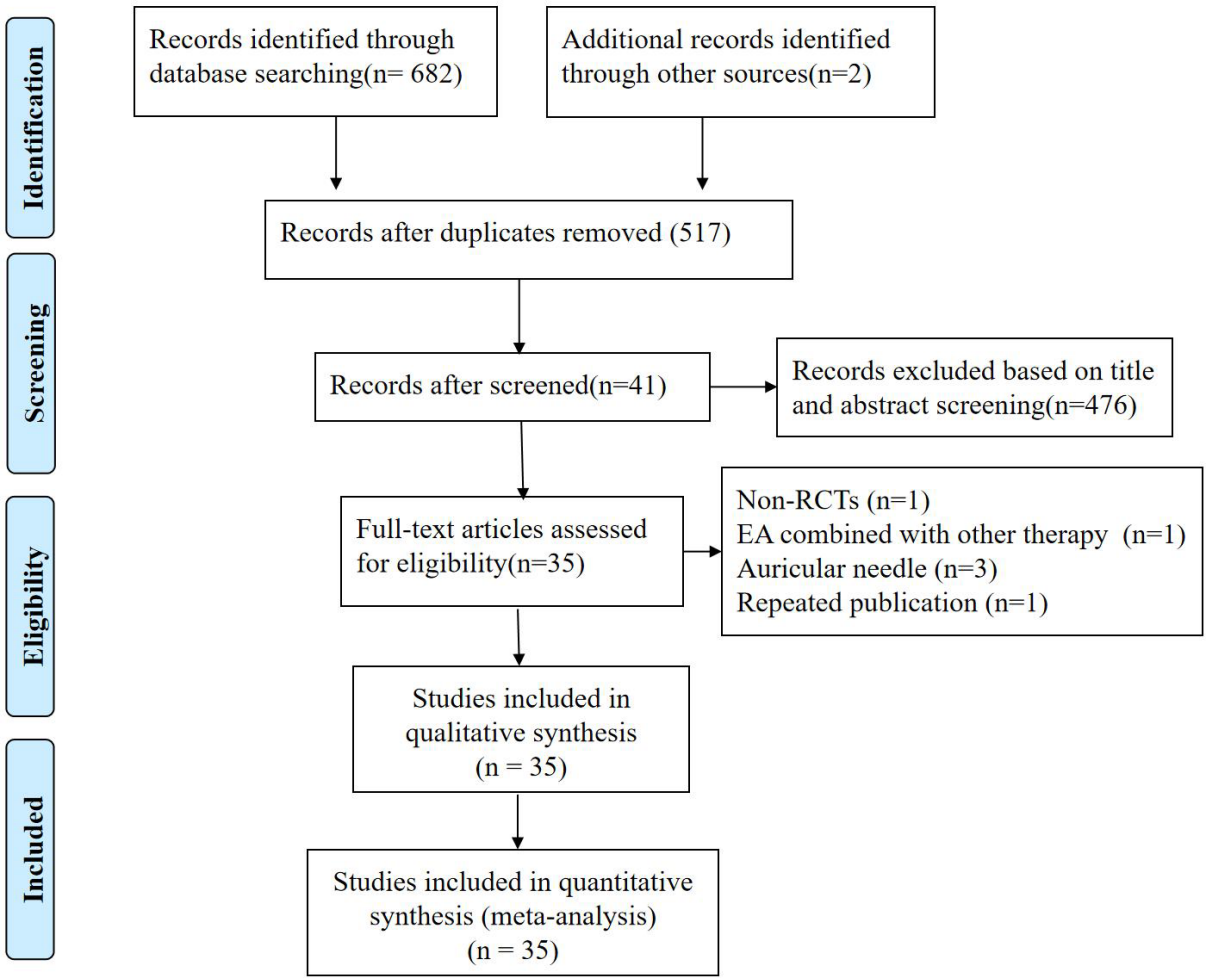
Flowchart of study selection.

Totally 684 potentially relevant strings were identified after a systematic search from the 7 databases. We removed 167 duplicates, and preliminarily eliminated 476 studies through screening titles and abstracts one by one. Among the remaining 41 studies, 6 studies were excluded by reading full text. Finally, 35 RCTs (15–49) with sample sizes ranging from 12 to 160 were fulfilled our inclusion criteria, which involving 3960 patients. Flow diagram of the screening process was illustrated in **Figure 1**.

### 3.2 Study characteristics

The main characteristics of the included RCTs were showed in **Supplementary eTable 1**. Four (15–17, 40) of the included studies examined the effectiveness of AA in contrast to placebo. Two (16, 17) among the 36 studies were designed as single-blind RCTs, and one (15) of them was a double-blind RCT. Treatment courses of the included studies ranged from one to six menstrual cycles, while the treatment lasted three menstrual cycles in the most of them (n=20). The first six auricular acupoints selected in the included studies were Internal Genitals (TF2, n=36), Endocrine (CO18, n=34), Shenmen (TF4, n=27), Liver (CO12, n=24), Sympathetic (AH6a, n=23), and Kidney (CO10, n=23).

### 3.3 Risk of bias

**Figure 2:**
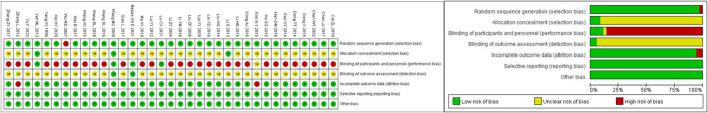
Risk of bias graph.

“Randomized” were described in all the included studies, while one study (41) reported an inappropriate method for sequence generation and was rated as “high risk”. Three of the included RCTs (15, 16, 31) were conducted with concealed allocation, and they were judged “low risk” for allocation concealment. The most included RCTs were judged “high risk” for blinding of participants and personnel because acupuncturists and participants could not be blinded, except for four studies (23, 24, 31, 40) using a placebo intervention. Two studies (18, 43) reported with a more than 15% dropout rate, so their “incomplete outcome data” were assessed as “high risk”. The detailed results are depicted in **Figure 2**.

### 3.4 Meta-analysis

#### 3.4.1 Primary outcomes

##### 3.4.1.1 Cured rate

**Figure 3:**
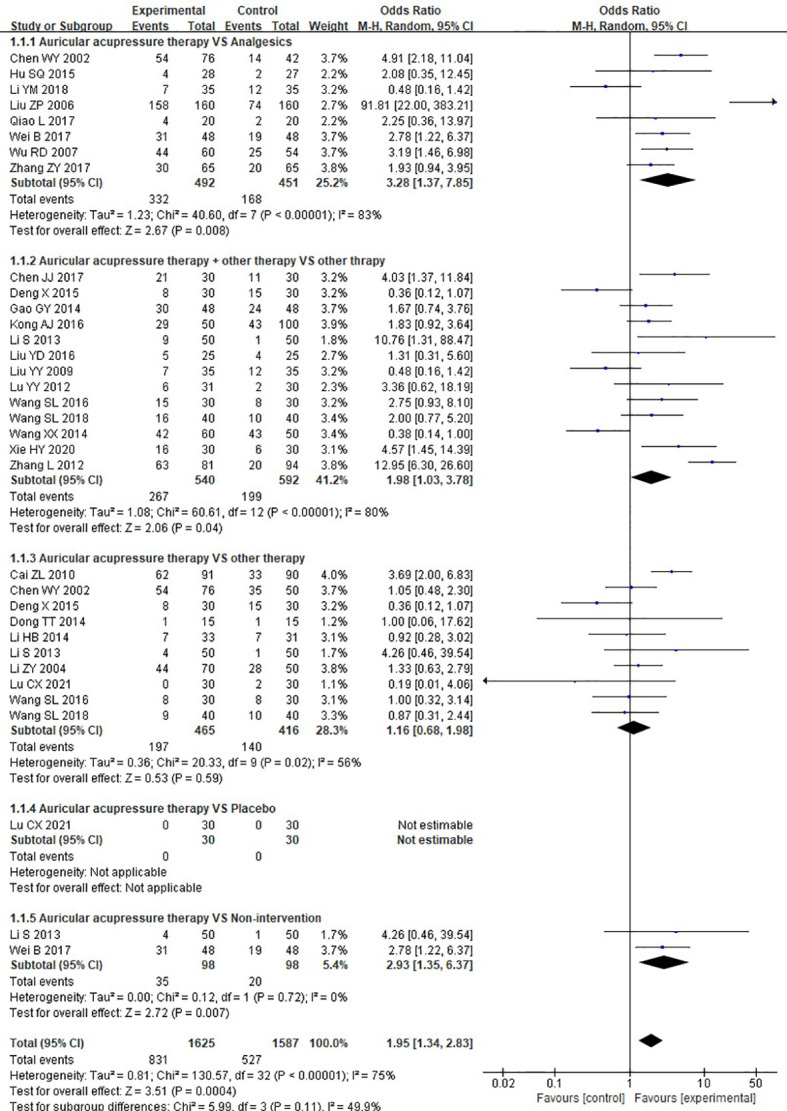
Meta-analysis and forest plot for cured rate.

Twenty-six RCTs reported data regarding cured rate. Meta-analysis results indicated that, overall, AA was associated with a significant benefit in cured rate (OR = 1.95, 95%CI: [1.34, 2.83], P=0.0004, I^2^ = 75%). After subgroup analysis, we found that AA showed a significantly higher cured rate when compared to analgesics (OR = 3.28, 95%CI: [1.37, 7.85], P=0.008, I^2^ = 83%) and non-intervention (OR = 2.93, 95%CI: [1.35, 6.37], P=0.007, I^2^ = 0%). Moreover, a significantly greater cured rate was observed in patients receiving AA combined with other therapy compared to those treated with other therapy alone (OR = 1.98, 95%CI: [1.03, 3.78], P=0.04, I^2^ = 80%), whereas no significant difference was detected when AA alone compared to other therapy (OR = 1.16, 95%CI: [0.68, 1.98], P=0.59, I^2^ = 56%). Only one study examined the efficacy of AA compared to placebo so that meta-analysis was not applicable, and no cured case was reported in the two groups in the study (**Figure 3**). Additionally, no difference was found in subgroup analysis comparing AA to Chinese herb (OR = 1.64, 95%CI: [0.63, 4.30], P=0.31, I^2^ = 75%), acupuncture (OR = 0.41, 95%CI: [0.15, 1.13], P=0.09, I^2^ = 0%), external application of Chinese herbal medicine (OR = 0.93, 95%CI: [0.43, 1.99], P=0.85, I^2^ = 0%), moxibustion (OR = 0.48, 95%CI: [0.04, 5.52], P=0.55, I^2^ = 77%), auricular needle (OR = 0.19, 95%CI: [0.01, 4.06], P=0.29, I^2^ = Not appliable), or health education (OR = 4.26, 95%CI: [0.46, 39.54], P=0.20, I^2^ = Not appliable) (**Supplementary eFigure 2**).

##### 3.4.1.2 Total effective rate

**Figure 4:**
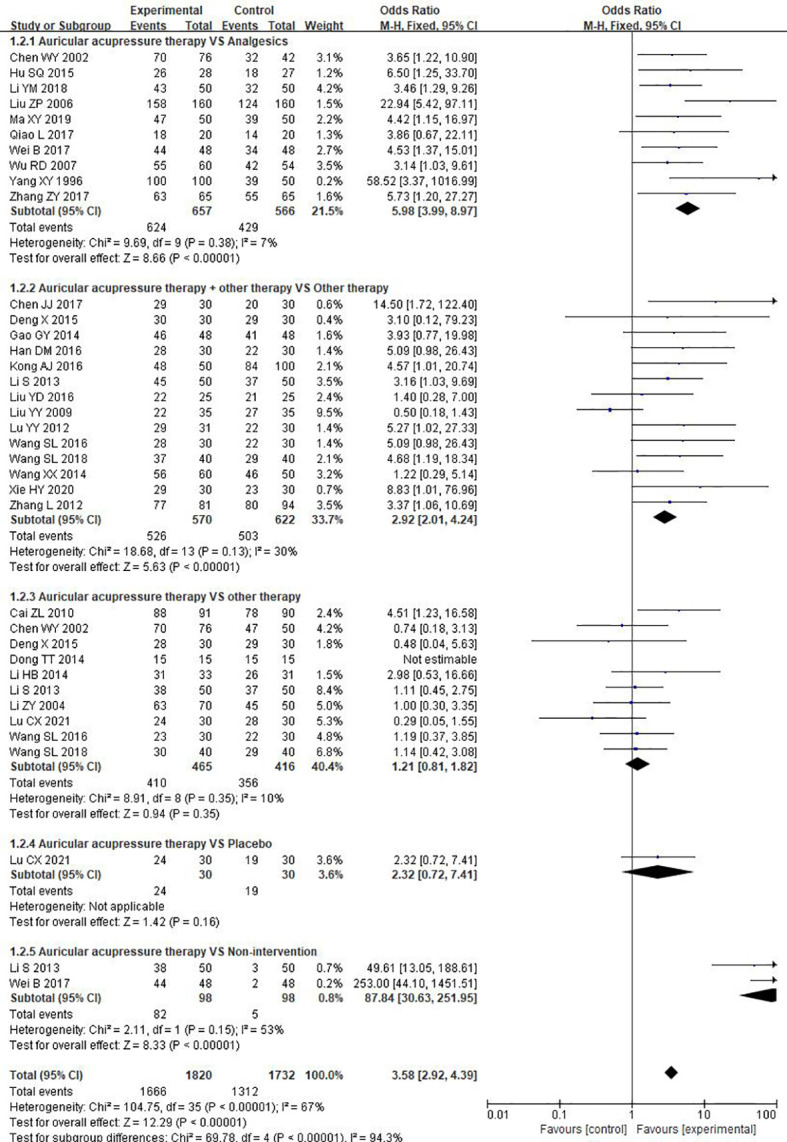
Meta-analysis and forest plot for total effective rate.

A total of twenty-eight RCTs reported data on total effective rate. Pooled data revealed that, overall, there was a significant increase in total effective rate (OR = 3.58, 95%CI: [2.92, 4.39], P<0.00001, I^2^ = 67%). A subgroup analysis was performed according to different comparators, and the results suggested that AA was superior to analgesics (OR = 5.98, 95%CI: [3.99, 8.97], P<0.00001, I^2^ = 7%) and non-intervention (OR = 87.84, 95%CI: [30.63, 251.95], P<0.00001, I^2^ = 53%). Moreover, AA combined with other therapy showed a higher total effective rate than other therapy used alone (OR = 2.92, 95%CI: [2.01, 4.24], P<0.00001, I^2^ = 30%). However, no significant difference in total effective rate was observed when AA used alone compared to other therapies (OR = 1.21, 95%CI: [0.81, 1.82], P=0.35, I^2^ = 10%) (**Figure 4**). Only one study described a close total effective rate by comparing AA and placebo (P=0.16), while meta-analysis could not be performed. Additionally, it was found in subgroup analysis that AA alone was not superior to Chinese herbs (OR = 2.17, 95%CI: [0.71, 6.67], P=0.18, I^2^ = 43%), acupuncture (OR = 0.48, 95%CI: [0.04, 5.63], P=0.56, I^2^ = Not appliable), external application of Chineseherbal medicine (OR = 1.16, 95%CI: [0.54, 2.48], P=0.70, I^2^ = 0%), moxibustion (OR = 1.00, 95%CI: [0.30, 3.35], P=1.00, I^2^ = Not appliable), auricular needle (OR = 0.29, 95%CI: [0.05, 1.55], P=0.15, I^2^ = Not appliable), or health education (OR = 1.11, 95%CI: [0.45, 2.75], P=0.82, I^2^ = Not appliable) (**Supplementary eFigure 3**).

##### 3.4.1.3 Visual analogue scale

**Figure 5:**
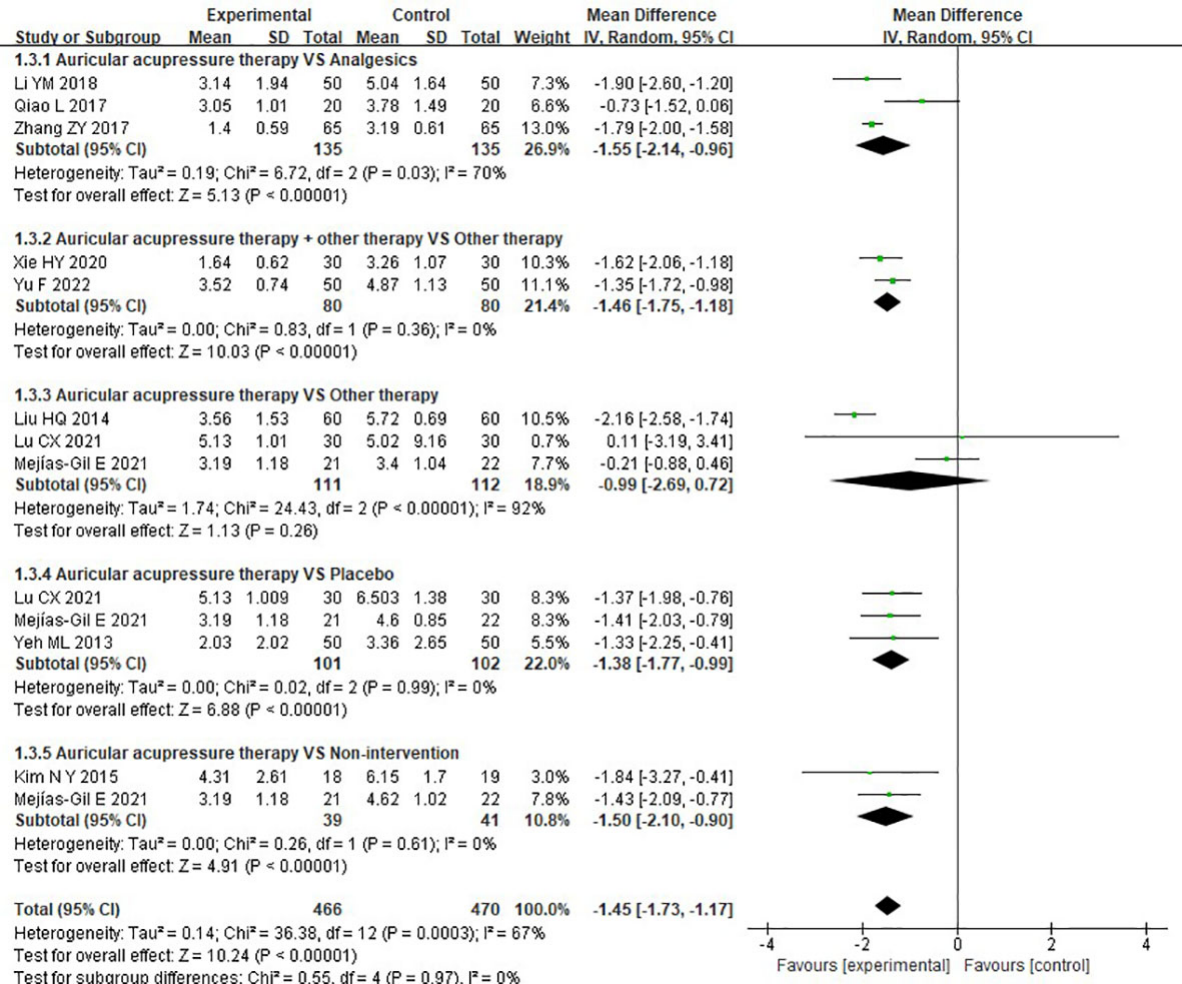
Meta-analysis and forest plot for VAS score.

Ten of the studies reported the effects of AA on VAS. Pooled results indicated that, overall, a significantly lower VAS score was observed in the experimental group than that in the control group (MD = -1.45, 95%CI: [-1.73, -1.17], P<0.00001, I^2^ = 67%). Furthermore, there was a significant reduction in VAS score in the experimental group when compared to analgesics (MD = -1.55, 95%CI: [-2.14, -0.96], P<0.00001, I^2^ = 70%), placebo (MD = -1.38, 95%CI: [-1.77, -0.99], P<0.00001, I^2^ = 0%), and non-intervention (MD = -1.50, 95%CI: [-2.10, -0.90], P=0.01, I^2^ = not appliable). And AA, as an adjunctive therapy to other therapy, also showed a lower VAS score than other therapy used alone (MD = -1.46, 95%CI: [-1.75, -1.18], P<0.00001, I^2^ = 0%). However, there was no significant difference in VAS score when AA alone compared to other therapy (MD = -0.99, 95%CI: [-2.69, 0.72], P=0.26, I^2^ = 92%) (**Figure 5**).

#### 3.4.2 Secondary outcomes

##### 3.4.2.1 Short-form menstrual distress questionnaire

**Figure 6:**
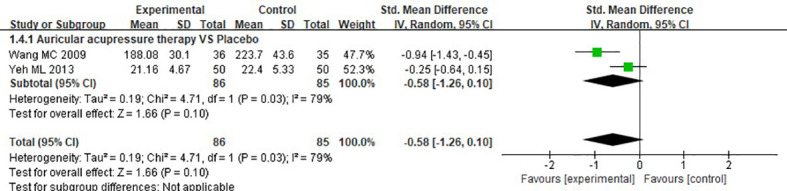
Meta-analysis and forest plot for MDQs.

Meta-analysis of two studies regarding MDQs showed that no significant difference in MDQs was found between the experimental group and control group (SMD = -0.58, 95%CI: [-1.26, 0.10], P= 0.10, I^2^ = 79%), (**Figure 6**).

##### 3.4.2.2 Symptom scores

**Figure 7:**
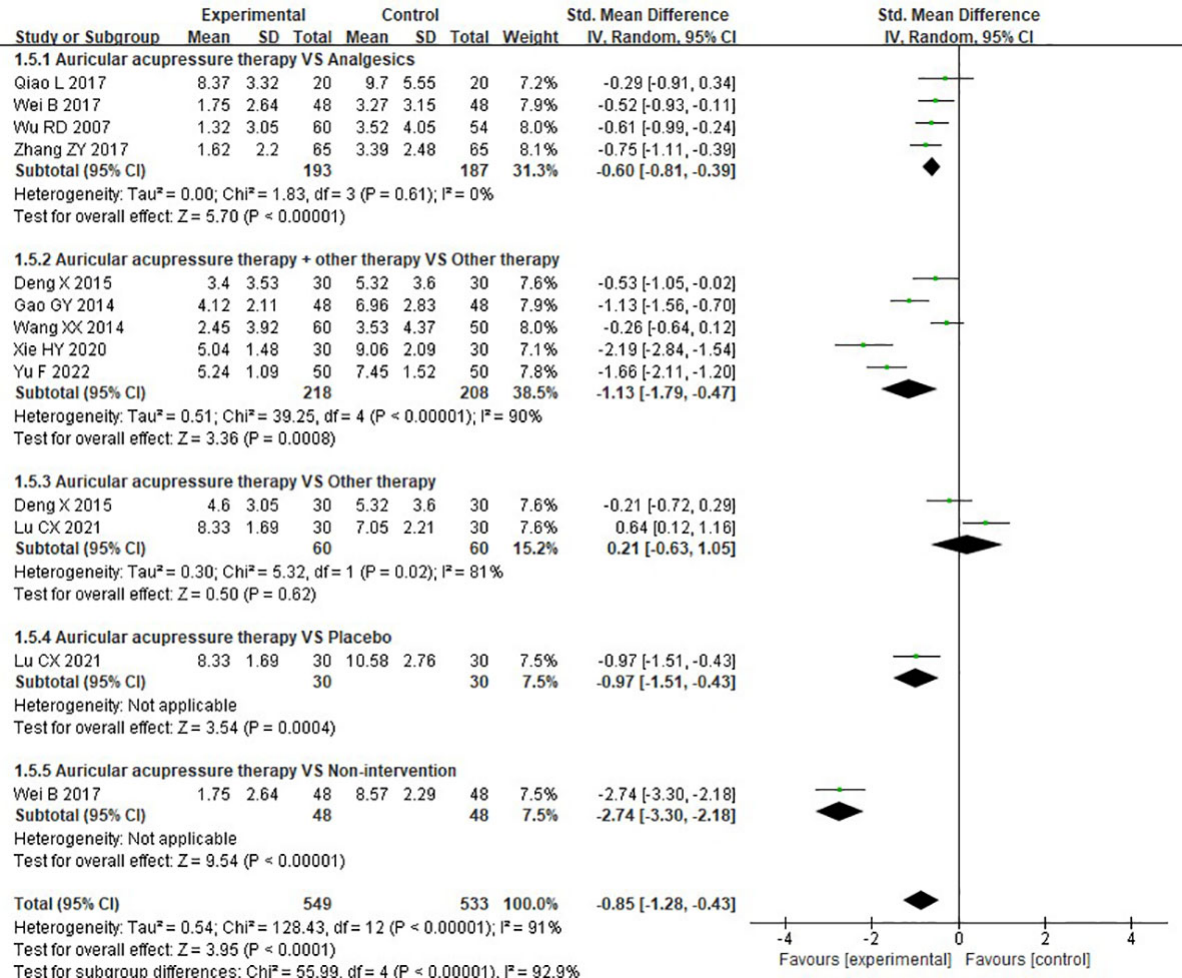
Meta-analysis and forest plot for symptom scores.

Eleven RCTs investigated the effects of AA on symptom scores. Meta-analysis result revealed that, overall, experimental group presented a greater reduction in symptom scores compared to the control group (SMD = -0.85, 95%CI: [-1.28, -0.43], P<0.0001, I^2^ = 91%). Results of subgroup analysis indicated that AA showed a significant improvement in symptom scores when compared to analgesics (SMD = -0.60, 95%CI: [-0.81, -0.39], P<0.00001, I^2^ = 0%), placebo (SMD = -0.97, 95%CI: [-1.51, -0.43], P=0.0004, I^2^ = not applicable), and non-intervention (SMD = -2.74, 95%CI: [-3.30, -2.18], P<0.0001, I^2^ = not applicable). Moreover, AA plus other therapy significantly improving symptom scores compared to other therapy used alone (SMD = -1.13, 95%CI: [-1.79, -0.47], P=0.0008, I^2^ = 90), while no difference in symptom scores between the patients receiving AA and other therapy alone (SMD = 0.21, 95%CI: [-0.63, 1.05], P=0.62, I^2^ = 81%) (**Figure 7**).

##### 3.4.2.3 Serum nitric oxide level

**Figure 8:**
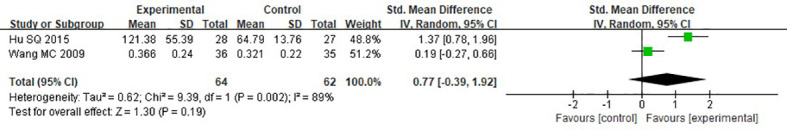
Meta-analysis and forest plot for Serum NO level.

Two RCTs reported the serum NO level. Meta-analysis results suggested no difference in serum NO level was determined in the experimental group than that in the control group (SMD = 0.77, 95%CI: [-0.39, 1.92], P =0.19, I^2^ = 89%) (**Figure 8**).

#### 3.4.3 Adverse events

One study (20) assessed the safety by laboratory examination, and recorded that there was no adverse event occurred during the treatment courses both in the experimental group and control group. Adverse event was not mentioned in the other studies.

#### 3.4.4 Publication bias

To assess the publication bias for primary and secondary outcomes, Begg’s test and Egger’ s test were applied in this study. As depicted in **Supplementary eTable 2**, we found that there could be publication bias for total effective rate by using Begg’s test (P=0.018). The remain P values calculated by using Begg’s test were more than 0.05, which indicated that the publication bias was not evident.

## 4 Discussions

Despite dysmenorrhea is considered to be a problem that plagues most women and girls, they show resistance and unwillingness to take medications. To resolve this clinical concern, large numbers of studies were conducted in order to explore an effective and simple approach. It has been suggested in previous studies that AA exerts a great impact on improving dysmenorrhea (50), while relevant evidence is still scarce. To the best of our knowledge, this is first systematical review and meta-analysis to determine whether AA was effective for the treatment of dysmenorrhea.

The findings of the present meta-analysis suggested that, overall, AA could significantly increase cured rate and total effective rate, decrease VAS score and symptom scores, which provided evidence that AA would be an effective method to improve symptoms for patients suffered from dysmenorrhea. Nevertheless, patients receiving AA showed no significant improvement in MDQs and serum NO level compared to those in the control list. Results of subgroup analysis based on different comparators, we found that AA was superior to analgesics, including Indomethacin (19, 28, 32, 35, 41, 42, 49), Ibuprofen (25, 35), Fenbid (42), Atropine (32), and Lumina (32), and non-invention for patients with dysmenorrhea in cured rate, total effective rate, VAS score, and symptom scores. It could be explained that analgesics only showed temporary improvement in pain symptoms, which was not fundamentally beneficial to prevent and treat dysmenorrhea. Meanwhile, AA presented positive effects on dysmenorrhea due to its regulation of qi and blood, and Yin and Yang. However, AA showed no significant improvement in cured rate, total effective rate, VAS score, and symptom scores compared to other therapies, including Chinese herb, acupuncture, external application of Chineseherbal medicine, moxibustion, auricular needle, and health education. Due to the temporary effect of analgesics on pain reduction, it would recur at the next menstrual cycle. Dissimilarly, AA alleviated pain through adjusting internal pathological state, such as rebalancing Yin and Yang, replenishing qi and blood and so on, to achieve fundamental pain improvement. Therefore, AA presented greater benefits in cured rate, total effective rate, and pain reduction than analgesics, while the other therapies, except for analgesics, also exhibited the same adjustive effects as AA, hence no difference in efficacy and symptomatic improvement was detected between them for dysmenorrhea patients.

Our findings revealed that a significantly higher total effective rate and cured rate were observed, when patients treated with AA combined with other therapy compared to those receiving AA alone. Moreover, the effect size of total effective rate was greater than that of the cured rate. It might be the reason that effective cases were more than cured cases in the two groups, which would result in a more detectable and significant statistical difference in total effective rate than cured rate. Notably, placebo control was set in the 4 studies, of which 2 performed with no seed (15, 17), one conducted with sham-needle (40) and the another used sham-acupoint (16). The meta-analysis for VAS scores indicated that patients receiving AA showed a significant lower VAS score than those treated with placebo. However, meta-analysis of two studies (13, 14) demonstrated that AA showed no significant reduction in MDQs scores when compared to placebo, and a similar result of total effective rate between AA and placebo was reported in only one study (40). Among included studies, the number of studies comparing the effects of AA to placebo was smaller, which would lead to a limited result. Additionally, meta-analysis results demonstrated that AA did not significantly adjust serum NO levels so studies on the mechanisms of AA on dysmenorrhea were needed. With respect to safety, it was only reported in one included study and no adverse event was described. Because AA was a non-invasive and non-pharmacological treatment, it caused few adverse events. Additionally, the factors for dysmenorrhea were variable, and the pathologies were also different between primary and secondary dysmenorrhea. In the present study, we included the trials regarding the two types of dysmenorrhea. Hence, it would be better to conduct studies to identify the effects of AA for dysmenorrhea with different pathologies in the future.

There are several limitations. Firstly, the auricular acupoints, times, and courses were selected without a consolidated standard in the included studies, which could cause clinical heterogeneities. Secondly, girls and women of reproductive age were enrolled in the most included studies, so it was difficult to exclude the individual factors at the patient level. Thirdly, random and blind methods were rarely reported in the included studies, which would contribute to risks of bias. Fourthly, there were only 4 trials regarding the comparator between AA and non-intervention, which led to a weak support to AA. Fifthly, most of the included studies reported in Chinese, hence there might be a language bias. Given the limitations of this study, future studies should be carried out strictly based on standard reporting guidelines such as CONSORT and more RCTs should focus on evaluating the therapeutic effects of AA on patients with dysmenorrhea compared to placebo.

## 5 Conclusions

The encouraging evidence of this study indicates that, overall, AA is an effective and potential safety therapy for the management of dysmenorrhea, including increasing cured rate and total effective rate, and improving VAS, and symptom scores. Nevertheless, AA showed no significant improvement in serum NO and MDQs. After subgroup analysis by different comparators, it is furtherly found that AA used alone is superior to analgesics and non-intervention regarding cured rate, total effective rate, VAS, and symptom scores. Furthermore, the same superiorities are observed when AA serves as an adjunctive strategy to other therapy. However, AA alone has little effect on them compared to other therapies, and there is no definite conclusion on the benefits of AA compared to placebo for patients with dysmenorrhea. Rigorous RCTs designed with blinded and sham-controlled are warranted to confirm these findings.

## Data availability statement

The original contributions presented in the study are included in the article/**Supplementary Material**. Further inquiries can be directed to the corresponding author.

## Author contributions

XK and YG designed the study. XK and HF performed the literature searches. XK and HF selected the studies. XK and HF extracted the data. XL and YZ completed the statistical analyses. XL, YZ, and YG revised the manuscript. All authors contributed to the article and approved the submitted version.
