# Supplementary material for: Effects of auricular acupressure on dysmenorrhea: A systematic review and meta-analysis of randomized controlled trials
Source: Front Endocrinol (Lausanne). 2023 Jan 5;13:1016222. doi: 10.3389/fendo.2022.1016222 (PMC9851274; doi:10.3389/fendo.2022.1016222)
Supplement: Supplementary file 1 [file DataSheet_1.doc]

**Supplementary materials**

**Pubmed**

**((Dysmenorrheas OR Pain, Menstrual OR Menstrual Pain OR Menstrual Pains OR Pains, Menstrual OR Menstruation, Painful OR Menstruations, Painful OR Painful Menstruation OR Painful Menstruations) AND (randomized controlled trial [Publication Type] OR randomized [Title/Abstract] OR placebo [Title/Abstract])) AND (auricular acupressure OR auriculotherapy OR Acupunctures, Ear OR Ear Acupunctures OR Auricular Acupuncture OR Ear Acupuncture OR Acupuncture, Auricular OR Acupunctures, Auricular OR Auricular Acupunctures OR Shiatsu OR Zhi Ya OR Chih Ya OR Shiatzu Acupuncture Points OR Pressure) 121**

**Embase**

| **No.** | **Query** | **Results** |
| --- | --- | --- |
| **#1** | **'dysmenorrhea'/exp** | **14,495** |
| **#2** | **'dysmenorrhea' OR 'dysmenorrheas' OR 'pain, menstrual' OR 'menstrual pain' OR 'menstrual pains' OR 'pains, menstrual' OR 'menstruation, painful' OR 'menstruations, painful' OR 'painful menstruation' OR 'painful menstruations'** | **16,243** |
| **#3** | **'auricular acupressure'/exp** | **45** |
| **#4** | **'auricular acupressure' OR 'acupunctures, ear' OR 'ear acupunctures' OR 'auricular acupuncture' OR 'ear acupuncture' OR 'acupuncture, auricular' OR 'acupunctures, auricular' OR 'auricular acupunctures' OR 'shiatsu' OR 'zhi ya' OR 'chih ya' OR 'shiatzu acupuncture points' OR 'pressure'** | **1,585,476** |
| **#5** | **'randomized controlled trial'/exp OR 'controlled clinical trial'/exp OR 'randomized':ti,ab OR 'placebo':ti,ab OR 'drug therapy':lnk OR 'randomly':ti,ab OR 'trial':ti,ab OR 'groups':ti,ab** | **9,012,077** |
| **#6** | **#1 OR #2** | **16243** |
| **#7** | **#3 OR #4** | **1585476** |
| **#8** | **#5 AND #6 AND #7** | **318** |

**Cochran**

| **ID** | **Search** | **Hits** |
| --- | --- | --- |
| **#1** | **MeSH descriptor: [Dysmenorrhea] explode all trees** | **704** |
| **#2** | **(Dysmenorrheas OR Pain, Menstrual OR Menstrual Pain OR Menstrual Pains OR Pains, Menstrual OR Menstruation, Painful OR Menstruations, Painful OR Painful Menstruation OR Painful Menstruations):ti,ab,kw (Word variations have been searched)** | **3868** |
| **#3** | **(auricular acupressure OR auriculotherapy OR Acupunctures, Ear OR Ear Acupunctures OR Auricular Acupuncture OR Ear Acupuncture OR Acupuncture, Auricular OR Acupunctures, Auricular OR Auricular Acupunctures OR Shiatsu OR Zhi Ya OR Chih Ya OR Shiatzu Acupuncture Points OR Pressure):ti,ab,kw (Word variations have been searched)** | **164922** |
| **#4** | **MeSH descriptor: [Acupuncture, Ear] explode all trees** | **216** |
| **#5** | **#1 or #2** | **3868** |
| **#6** | **#3 or #4** | **164922** |
| **#7** | **#5 and #6** | **298** |

**CNKI**

**( SU %= '耳穴' OR TKA = '耳穴' OR TKA = '耳穴压籽' OR TKA = '耳穴压豆' OR TKA = '耳穴压丸' OR TKA = '耳穴贴压' OR TKA = '耳穴埋豆' OR TKA = '耳穴埋籽' OR TKA = '耳穴埋丸' OR TKA = '耳穴疗法') AND ( TKA = '痛经' OR SU %= '痛经' OR TKA = '经期综合征' OR TKA = '经期疼痛' OR TKA = '经期综合症' ) AND ( TKA = '对照' OR TKA = '随机') 159**

**Wangfang Database**

**(主题:(耳穴) or 主题:(耳穴压籽) or 主题:(耳穴压豆) or 主题:(耳穴压丸) or 主题:(耳穴贴压) or 主题:(耳穴埋豆) or 主题:(耳穴埋籽) or 主题:(耳穴埋丸) or 主题:(耳穴疗法) ) and (主题:(痛经) or 主题:(经期综合征) or 主题:(经期疼痛) or 主题:(经期综合症) ) and (主题:(对照) or 主题:(随机) ) 128**

**CQVIP Database**

**( R=耳穴 OR R=耳穴 OR R=耳穴压籽 OR R=耳穴压豆 OR R=耳穴压丸 OR R=耳穴贴压 OR R=耳穴埋豆 OR R=耳穴埋籽 OR R=耳穴埋丸 OR R=耳穴疗法) AND (R=痛经 OR R=痛经 OR R=经期综合征 OR R=经期疼痛 OR R=经期综合症) AND (R=对照 OR R=随机) 81**

**Supplementary eFigure 1** The search strategy in this review.


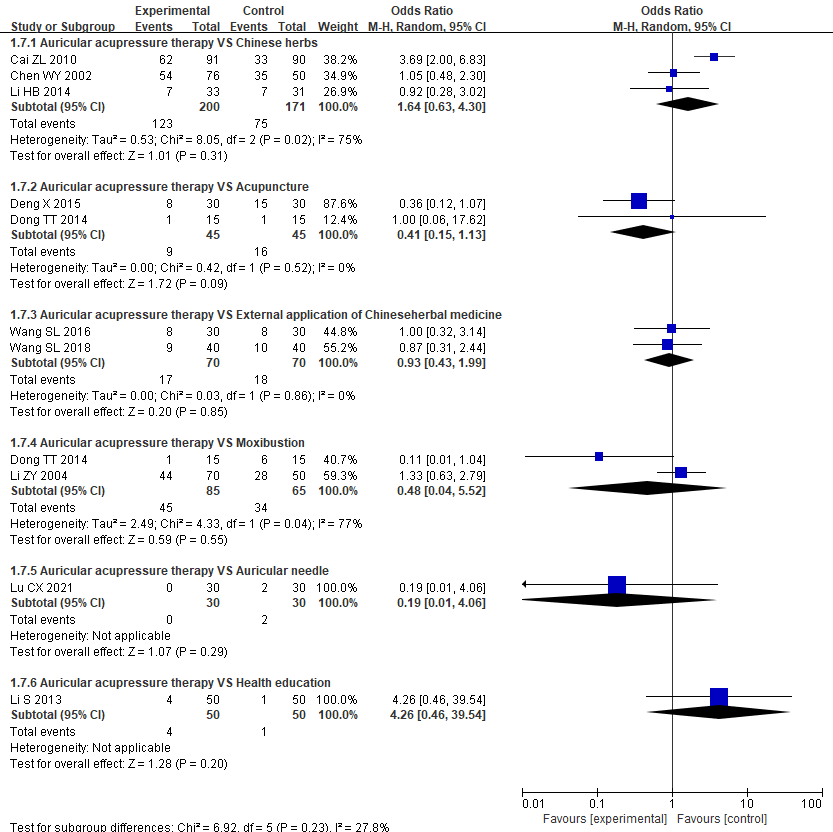


**Supplementary eFigure 2** Subgroup analysis of cured rate for Aucicular acupressure VS other therapies (e.g. Chinese herb, acupuncture, external application of Chineseherbal medicine, moxibustion, aucicular needle, and health education)


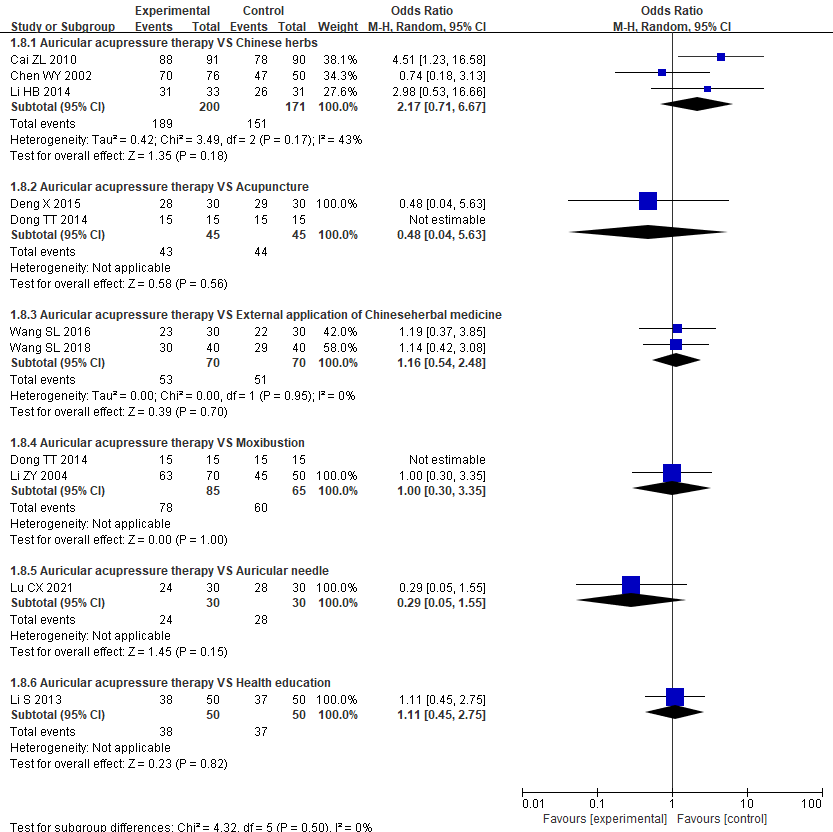


**Supplementary eFigure 3** Subgroup analysis of total effective rate for Aucicular acupressure VS other therapies (e.g. Chinese herb, acupuncture, external application of Chineseherbal medicine, moxibustion, aucicular needle, and health education)

**Supplementary eTable 1 Characteristics of the included studies.**

| No. | First author (Year) | Country | study  design | auricular acupressure points | Intervention parameters | | Intensity | Main outcome |
| --- | --- | --- | --- | --- | --- | --- | --- | --- |
| 1 | Wang MC 2009^15^ | China (Taiwan) | RCT | Liver (CO12), Kidney (CO10), and Endocrine (CO18) | Group 1 (n=36, years: 22.3±2.4): Semen vaccariae seeds were sticked on the acupressure points by using adhesive plasters, and manually pressed 15 times on each point, 3 times a day for 20 days;  Group 2 (n=35, years: 22.6±2.6): preformed with no seed. The adhesive patch with/without the ear seed was renewed every 5 days. | Manually pressing lightly on every acupressure  point | | MDQs, NO |
| 2 | Yeh ML 2013^16^ | China (Taiwan) | RCT | shenmen (TF4), Internal Genitals (TF2), Central Rim (AT2.3.4i), Liver (CO12), Kidney (CO10), and Endocrine (CO18) | Group 1 (n=50, years: 17.94±0.84): cowherb seeds were sticked on the acupressure points by using plasters, and pressed on each acupoint for 1 minute, 4 times a day for 2 days from the onset of menstrual pain;  Group 2 (n=50, years: 17.78±0.89): preformed on sham acupoints including Wind Stream, Esophagus, Trachea, Pharynx and Larynx, Internal Nose, and Tonsil. | NR | | MDQs, VAS |
| 3 | Mejías-Gil E 2021^17^ | Spain | RCT | Shenmen (TF4), Internal Genitals (FT2), sympathetic (AH6a), kidney (CO10), heart (CO15), endocrine (CO18) and thalamus (PC2). | Group 1 (n=21, years: 20.95±1.85): vaccariae seeds were sticked on the acupressure points by using plasters, and manually pressed within 4h from the beginning of the menstrual cycle and were maintained during 72h. The treatment was conducted during 4 menstrual cycles.  Group 2 (n=22, years: 20.64 ± 1.05): kinesio-tape group. the dosage and course were the same as those of Group 1.  Group 3 (n=22, years: 21.14 ± 0.99): preformed with no seed. the dosage and course were the same as those of Group 1.  Group 4 (n=22, years: 20.91 ± 1.26): non-intervention.  Group 5 (n=21, years: 20.95 ± 1.32): kinesio-tape placebo group. | NR | | VAS |
| 4 | Kim NY 2015^18^ | Korea | RCT | Shenmen (TF4), pelvic cavity (TF5), adrenal gland (TG2p), Internal Genitals (FT2), sympathetic (AH6a), kidney (CO10), heart (CO15), spleen (CO13), stomach (CO4), endocrine [CO18], thalamus (PC2), occiput (AT3), subcortex (AT4), center of superior concha, ear apex, ovary, and adnexa. | Group 1 (n = 12, years: 21.92 ± 4.78): Energy-move Stones were sticked on the acupressure points by using plasters, and manually pressed on the first or second day of their menstrual period.  Group 2 (n= 18, years: 21.39±1.72): Energy-move Stones were sticked on the acupressure points by using plasters, and manually pressed once a week for a month.  Group 3 (n= 19, years: 22.21 ± 3.08): received no auricular acupressure therapy. | NR | | VAS |
| 5 | Zhang ZY 2017^19^ | China | RCT | Liver (CO12), shenmen (TF4), Internal Genitals (TF2), and Endocrine (CO18). | Group 1 (n=65, years: 22.3±1.5): Semen vaccariae seeds were sticked on the acupressure points by using adhesive plasters from one side to another every 2 days, and manually pressed 5 minutes from 7 days before the beginning of the menstrual cycle to 7 days after menstruation for 3 menstrual cycles;  Group 2 (n=65, years: 22.2 ± 1.4): treated with indomethacin enteric coated tablets orally during menstruation, 25 mg twice a day until the pain disappears for 3 menstrual cycles. | Press with the finger pulp, from light to heavy until the finger pulp is swollen. | | symptom scores, VAS, cured rate, total effective rate |
| 6 | Han DM 2016^20^ | China | RCT | Shenmen (TF4), Internal Genitals (TF2), sympathesis (AH6a), Liver (CO12), Kidney (CO10), Endocrine (CO18), and subcortex (AT4). | Group 1 (n=30, years: 20 ± 3): based on the treatment with a decoction of medicinal ingredients (Take 150 mL orally each 30 minutes after breakfast and dinner for 7 days before each menstruation), Semen vaccariae seeds were sticked on the acupressure points by using adhesive plasters, and manually pressed 3 ～ 5 times a day starting from 3 ～ 5 days before the beginning of the menstrual cycle for 7 days. The course of treatment lasted for 3 menstrual cycles;  Group 2 (n=30, years: 19 ± 4): treated with a decoction of medicinal ingredients only, and the dosage and course were the same as those of Group 1. | Finger press to the patient's maximum tolerance. | | cured rate, total effective rate |
| 7 | Cai ZL 2010^21^ | China | RCT | shenmen (TF4), Internal Genitals (TF2), sympathesis (AH6a), Liver (CO12), Kidney (CO10), Endocrine (CO18), subcortex (AT4), pelvic cavity (TF5), and abdomen (AH8). | Group 1 (n=91, years: 17～42): Semen vaccariae seeds were sticked on the acupressure points by using adhesive plasters from one side to another every other day, and manually pressed 5 minutes every 2 hours for 7 days starting from the beginning of the menstrual cycle;  Group 2 (n=90, years: 18～41): treated with Chinese patent medicine (Tongjingbao granule, national drug approval number: Z41021972) orally during menstruation, 1 bag for a once, 3 times a day for 7 days. | NR | | cured rate, total effective rate |
| 8 | Xie HY 2020^22^ | China | RCT | Shenmen (TF4), Internal Genitals (TF2), sympathesis (AH6a), Liver (CO12), Kidney (CO10), Endocrine (CO18), and subcortex (AT4). | Group 1 (n=30, years: 24. 06 ± 3. 21): based on the treatment with Shaofu Zhuyu decoction orally (150 mL for a once, 2 times a day) from the beginning to the end of the menstrual cycle, Semen vaccariae seeds were sticked on the acupressure points by using adhesive plasters from one side to another every 3 days, and manually pressed 60 seconds for a once, 3 times a day for 3 menstrual cycles;  Group 2 (n=30, years: 25. 11 ± 3. 14): treated with Shaofu Zhuyu decoction orally from the beginning to the end of the menstrual cycle, 150 mL for a once, 2 times a day for 3 menstrual cycles. | Finger pressing to local slight pain and fever, numbness, or the maximum tolerance. | | TCM symptom scores, VAS, cured rate, total effective rate |
| 9 | Yang XY 1996^23^ | China | RCT | Internal Genitals (TF2), sympathesis (AH6a), subcortex (AT4), ovary, and Endocrine (CO18) | Group 1 (n=100, years: 16～42): Semen vaccariae seeds were sticked on the acupressure points by using plasters, and pressed on each acupoint for 1 minute, 4 times a day for 2 days from the onset of menstrual pain; The course of treatment lasted for 3 menstrual cycles;  Group 2 (n=50, years: 16～42): treated with analgesic for 3 menstrual cycles. | Press to swollen, numbness, or local slight pain and fever. | | total effective rate |
| 10 | Liu YY 2009^24^ | China | RCT | Internal Genitals (TF2), sympathesis (AH6a), subcortex (AT4), kidney [CO10], spleen (CO13), ovary, and Endocrine (CO18) | Group 1 (n = 35, years: 35.63±3.7): based on the treatment with acupuncture [acupoints: Taixi (KI3), Diji (SP8), Zigong (EX-CA1), Dahe (KI12), Guangyuan (RN4), and Qihai (RN6), 30 minutes a day for 7 days starting from 3 ～ 5 days before the beginning of the menstrual cycle], Semen vaccariae seeds were sticked on the acupressure points by using adhesive plasters from one side to another every other day, and manually pressed 10 ～ 20 minutes for a once, 4 times for 3 menstrual cycles;  Group 2 (n = 35, years: 35.63±3.7): treated with acupuncture. The acupoints, dosage and course were the same as those of Group 1.  Group 3 (n = 35, years: 35.63±3.7): treated with acupuncture and moxibustion, 30-minutes acupuncture and 20-minutes moxibustion a day for 7 days starting from 3 ～ 5 days before the beginning of the menstrual cycle | NR | | cured rate, total effective rate |
| 11 | Li YM 2018^25^ | China | RCT | Internal Genitals (TF2), sympathesis (AH6a), subcortex (AT4), ovary, Endocrine (CO18), and shenmen (TF4) | Group 1 (n=50, years: 31.2±3.4): Semen vaccariae seeds were sticked on the acupressure points by using adhesive plasters, and manually pressed 3 ～ 4 times a day from 7 days before the beginning of the menstrual cycle until the symptoms of dysmenorrhea were completely relieved, a total of 3 menstrual cycles;  Group 2 (n=50, years: 27.5±2.8): Took Ibuprofen sustained-release capsule orally on the first day of menstrual cycle until the symptoms of dysmenorrhea were completely relieved, 0.3g for a once, once a day, a total of 3 menstrual cycles. | Press to swollen, numbness, or local slight pain and fever. | | VAS, cured rate, total effective rate |
| 12 | Li ZY 2004^26^ | China | RCT | Internal Genitals (TF2), sympathesis (AH6a), Endocrine (CO18), and shenmen (TF4). | Group 1 (n=70, years: 14～35): Semen vaccariae seeds were sticked on the acupressure points by using adhesive plasters, and manually pressed the bilateral acupressure points until the symptoms of dysmenorrhea were completely relieved during menstruation. Meanwhile, during the non-menstruation, the seeds were sticke on the points from one side to another every 2～3 days, and participants were asked to manually pressed 1minutes, 3 ～ 5 times a day from 5 ～7 days before the beginning of the menstrual cycle to when it came, a total of 3 menstrual cycles;  Group 2 (n=50, years: 14～35): treated with warm moxibustion on lumbosacral region, 30 ～ 40 minutes for a once, once a day, a total of 3 ～ 5 times. | Press to swollen, numbness, or local slight pain and fever. | | cured rate, total effective rate |
| 13 | Deng X 2015^27^ | China | RCT | Shenmen (TF4), Internal Genitals (TF2), sympathesis (AH6a), Liver (CO12), Kidney (CO10), and Endocrine (CO18). | Group 1 (n=30, years: 18～23): treated with acupuncture; acupoints: Guanyuan (RN4), Zusanli (ST36), Sanyinjiao (SP6); 30 minutes per once, once a day, from 5 ～7 days before the beginning of the menstrual cycle to when it came, a total of 3 menstrual cycle.  Group 2 (n=30, years: 18～23): Semen vaccariae seeds were sticked on the acupressure points by using adhesive plasters from one side to another, and manually pressed every 2 hours, 3 ～ 5 times a day from the beginning to the end of the menstrual cycle. The course of treatment lasted for 3 menstrual cycles.  Group 3 (n=30, years: 18～23): a combination treatment was applied (accupuncture combined with auricular accupresure, and the treatment parameters were the same as the Group 1 and 2). | NR | | symptom scores, cured rate, total effective rate |
| 14 | Wei B 2017^28^ | China | RCT | Shenmen (TF4), Internal Genitals (TF2), Liver (CO12), and Endocrine (CO18). | Group 1 (n=48, years: 21 ± 1): Semen vaccariae seeds were sticked on the acupressure points by using adhesive plasters from one side to another every other day , and manually pressed 3 ～ 5 minutes a day from 7 days before the beginning of the menstrual cycle to 7 days after menstruation for 3 menstrual cycles;  Gropus 2 (n=48, years: 21 ± 1): took indomethacin enteric coated tablets orally, 25 mg for a once, twice a day from the beginning of the menstrual cycle until the symptoms of dysmenorrhea were completely relieved, a total of 3 menstrual cycles;  Group 3 (n=48, years: 21 ± 1): non-intervention. | Press with the finger pulp to swollen. | | symptom scores, cured rate, total effective rate |
| 15 | Wang SL 2018^29^ | China | RCT | Shenmen (TF4), Internal Genitals (TF2), sympathesis (AH6a), Liver (CO12), kidney (CO10), spleen (CO13), and Endocrine (CO18). | Group 1 (n=40, years: 23.30±2.14): treated with acupoint application of Chinese herb [acupoints: shenque (RN8),Guangyuan (RN4), and Qihai (RN6)], and auricular acupressure (Semen vaccariae seeds were sticked on the acupressure points by using adhesive plasters, and manually pressed 1 minutes for a once from 3 ～ 5 days before the beginning to the end of the menstrual cycle, 3 ～ 6 times a day, for 3 menstrual cycles);  Group 2 (n=40, years: 23.30±2.14): treated with acupoint application of Chinese herb alone;  Group 3 (n=40, years: 23.30±2.14): treated with auricular acupressure alone. | Press to swollen, numbness, or local slight pain and fever. | | cured rate, total effective rate |
| 16 | Li HB 2014^30^ | China | RCT | Shenmen (TF4), Internal Genitals (TF2), sympathesis (AH6a), Liver (CO12), Kidney (CO10), and Endocrine (CO18) | Group 1 (n=33, years: NR):Semen vaccariae seeds were sticked on the acupressure points by using adhesive plasters from one side to another every 3 days, and manually pressed 5 times a day 5 minutes from 3 ～ 5 days before the beginning of the menstrual cycle until the symptoms of dysmenorrhea were completely relieved, a total of 3 menstrual cycles;  Group 2 (n=31, years: NR): treated with Chinese patent medicine (Tianqi Tongjing Capusle, national drug approval number: Z44020926), 1.2 g for a once, twice a day from the beginning of the menstrual cycle until the symptoms of dysmenorrhea were completely relieved, a total of 3 menstrual cycles. | Press to swollen, numbness, or local slight pain and fever. | | symptom scores, cured rate, total effective rate |
| 17 | Li S 2013^31^ | China | RCT | Shenmen (TF4), Internal Genitals (TF2), sympathesis (AH6a), Liver (CO12), Kidney (CO10), and Endocrine (CO18) | Group 1 (n=50, years: 17 ～ 20): auricular acupressure (Semen vaccariae seeds were sticked on the acupressure points by using adhesive plasters, and manually pressed 3 ～ 5 times, 1 ～ 3 minutes for a once, for 3 menstrual cycles);  Gropus 2 (n=50, years: 17 ～ 20): health education;  Group 3 (n=50, years: 17 ～ 20): auricular acupressure + health education;  Group 4 (n=50, years:): non-intervention. | Press to swollen, numbness, or local slight pain and fever until the maximum tolerance. | | symptom scores, cured rate, total effective rate |
| 18 | Chen WY 2002^32^ | China | RCT | Internal Genitals (TF2), sympathesis (AH6a), ovary, pelvic cavity (TF5), and Endocrine (CO18) | Group 1 (n=76, years: 16 ～ 35): Semen vaccariae seeds were sticked on the acupressure points by using adhesive plasters from one side to another every other day, and manually pressed 3 ～ 5 minutes, 3 times a day from 2 ～ 3 days before the beginning of the menstrual cycle and lasting for 4 days, a total of 3 menstrual cycles;  Gropus 2 (n=50, years: 16 ～ 35): Chinese medicine decoction, one dose per day, taken twice for 4 days, a total of 3 menstrual cycles;  Gropus 3 (n=42, years: 16 ～ 35): treated with indomethacin, atropine, or lumina. | Press to swollen, numbness, or fever. | | cured rate, total effective rate |
| 19 | Chen JJ 2017^33^ | China | RCT | Shenmen (TF4), Internal Genitals (TF2), sympathesis (AH6a), Liver (CO12), and abdomen (AH8). | Group 1 (n=30, years: 14 ～ 30): Shaofu Zhuyu decoction was taken orally, one dose a day. Meanwhile, semen vaccariae seeds were sticked on the acupressure points by using adhesive plasters, and manually pressed 30 ～ 60 seconds, 3 ～ 5 times a day. The treatment started from 4 ～ 5 days before the beginning of the menstrual cycle and lasted for 5 days, a total of 6 menstrual cycles;  Group 2 (n=30, years: 14 ～ 30): Shaofu Zhuyu decoction was used alone. | NR | | cured rate, total effective rate |
| 20 | Dong TT 2014^34^ | China | RCT | Internal Genitals (TF2), ovary, Liver (CO12), Kidney (CO10), spleen (CO13), and Endocrine (CO18). | Group 1 (n=15, years: 18 ～ 23): treated wtih acupuncture [acupoints: Taichong (LR3), Guangyuan (RN4), Zusanli (ST36), Sanyinjiao (SP6), Diji (SP8), Xuehai (SP10), Shuidao (ST28), Hegu (LI4), 30 minutes for a once, once every other day, from 14 days before the beginning to the end of the menstrual cycle ays, a total of 3 menstrual cycles];  Group 2 (n=15, years: 18 ～ 23): treated with moxibustion [acupoints: Qihai (RN6), Sanyinjiao (SP6), Zhongji (RN3), Shenque (RN8), Shuidao (ST28), Taichong (LR3), Xuehai (SP10), 30 minutes for a once, once every other day, from 14 days before the beginning to the end of the menstrual cycle, a total of 3 menstrual cycles];  Group 3 (n=15, years: 18 ～ 23): Semen vaccariae seeds were sticked on the acupressure points by using adhesive plasters from one side to another every 3 days, and manually pressed from 14 days before the beginning to the end of the menstrual cycle, a total of 3 menstrual cycles. | NR | | cured rate, total effective rate |
| 21 | Hu SQ 2015^35^ | China | RCT | Internal Genitals (TF2), ovary, Liver (CO12), Kidney (CO10), spleen (CO13), and Endocrine (CO18). | Group 1 (n=28, years: 19.65±2.17): Semen vaccariae seeds were sticked on the acupressure points by using adhesive plasters, and manually pressed 3 ～ 5 times a day from 3 days before the beginning of the menstrual cycle until the symptoms of dysmenorrhea were completely relieved, a total of 3 menstrual cycles;  Group 2 (n=27, years: 19.87±2.09): treated with indomethacin enteric coated tablets or ibuprofen, taken according to the instructions, from 3 days before the beginning from the beginning of the menstrual cycle until the symptoms of dysmenorrhea were completely relieved, a total of 3 menstrual cycles. | Press to swollen, numbness, or local slight pain. | | NO, cured rate, total effective rate |
| 22 | Ma XY 2019^36^ | China | RCT | Shenmen (TF4), Internal Genitals (TF2), Liver (CO12), and Endocrine (CO18). | Group 1 (n=50, years: 20.4±1.6): Semen vaccariae seeds were sticked on the acupressure points by using adhesive plasters from one side to another every other day, and manually pressed 3 ～ 5 times a day from 7 days before the beginning of the menstrual cycle to 7 days after menstruation for 3 menstrual cycles;  Group 2 (n=50, years: 20.4±1.6): treated with indomethacin enteric coated tablets, 25 mg / once, twice a day, lasting for 7 days until the symptoms of dysmenorrhea were completely relieved, a total of 3 menstrual cycles. | Press to swollen, numbness, or local slight pain. | | cured rate, total effective rate |
| 23 | Liu HQ 2014^37^ | China | RCT | Shenmen (TF4), Internal Genitals (TF2), sympathesis (AH6a), uppertrgus (TG1), and Endocrine (CO18). | Group 1 (n=60, years: 28.16±7.34): Semen vaccariae seeds were sticked on the acupressure points by using adhesive plasters, and manually pressed 3 ～ 4 times a day from 7 days before the beginning of the menstrual cycle to 7 days after menstruation for 3 menstrual cycles;  Group 2 (n=60, years: 30.94±6.23): Yuanhu Zhitong tablet, taken 4 pills a day from the onset of menstrual pain until dysmenorrhea symptoms do not affect work and study, a total of 3 menstrual cycles. | Press to swollen, numbness, or local slight pain and fever. | | VAS |
| 24 | Lu YY 2012^38^ | China | RCT | Shenmen (TF4), Internal Genitals (TF2), sympathesis (AH6a), Liver (CO12), Kidney (CO10), and Endocrine (CO18) | Group 1 (n=31, years: 25.9±5.97): Based on treatments with accupuncture and moxibustion (30 minutes for a once, once/twice a day, 3 ～ 5 days until the symptoms of dysmenorrhea were completely relieved), Semen vaccariae seeds were sticked on the acupressure points by using adhesive plasters, and manually pressed each acupoint 50 times for a once, 3 ～ 4 times a day from 7 days before the beginning of the menstrual cycle to 7 days after menstruation. The course of treatment lasted for 6 menstrual cycles;  Group 2 (n=30, years: 25.5±5.67): treated with accupuncture and moxibustion, 30 minutes for a once, once/twice a day, 3 ～ 5 days until the symptoms of dysmenorrhea were completely relieved for 6 menstrual cycles. | Press to swollen, numbness, or local slight pain and fever. | | cured rate, total effective rate |
| 25 | Gao GY 2014^39^ | China | RCT | Shenmen (TF4), Internal Genitals (TF2), sympathesis (AH6a), Endocrine (CO18), subcortex (AT4), and pelvic cavity (TF5). | Group 1 (n=48, years: 19.2±6.6): treated with Duyiwei capusle (national drug approval number:Z10970053, taken orally, 3 pills /once, 3 times a day, from 3 days before the beginning of the menstrual cycle to 3 days after menstruation); meanwhile, semen vaccariae seeds were sticked on the acupressure points by using adhesive plasters from one side to another every 3 days, and manually pressed each acupoint 2 ～ 4 minutes for a once, 4 ～ 5 times a day for 6 days. The course of treatment lasted for 6 menstrual cycles.  Group 2 (n=48, years: 18.8±7.2): treated with Duyiwei capusle alone. The dosage and course were the same as those of EG. | Press to swollen, numbness, or local slight pain and fever. | | cured rate, total effective rate |
| 26 | Lu CX 2021^40^ | China | RCT | Shenmen (TF4), Internal Genitals (TF2), sympathesis (AH6a), Liver (CO12), Kidney (CO10), subcortex (AT4), and Endocrine (CO18) | Group 1 (n=30, years: 20 ～ 39): treated with auricular intradermal needling from one side to another every other day; pressing and kneading for 3 ～4 minutes per once, 3 ～4 times a day, 4 times each menstrual cycle, from 5 days before the beginning of the menstrual cycle for a total of 3 menstrual cycles.  Group 2 (n=30, years: 20 ～ 39): Semen vaccariae seeds were sticked on the acupressure points by using adhesive plasters from one side to another every other day, and manually pressed 3 ～ 4 minutes for a once, 3 ～ 4 times a day 4 times each menstrual cycle, from 5 days before the beginning of the menstrual cycle for a total of 3 menstrual cycles.  Group 3 (n=30, years: 20 ～ 39): placebo. | Press to swollen, and slight fever. | | VAS, symptom scores, cured rate, total effective rate |
| 27 | Wu RD 2007^41^ | China | RCT | Shenmen (TF4), Internal Genitals (TF2), sympathesis (AH6a), Kidney (CO10), subcortex (AT4), and Endocrine (CO19) | Group 1 (n=60, years: 15～25): Semen vaccariae seeds were sticked on the acupressure points by using adhesive plasters from one side to another every other day, and manually pressed 1 ～ 2 minutes for a once, 5 ～ 6 times a day, 4 times each menstrual cycle, from the onset of menstrual pain to 3 days after the symptoms of dysmenorrhea were completely relieved. The course of treatment lasted for 3 menstrual cycles.  Group 2 (n=54, years: 14～25): treated with oral administration of Indomeixin enteric tablets, 25 mg/ once, 3 times a day for 7 days. The course of treatment lasted for 3 menstrual cycles. | Press to swollen, numbness, or local slight pain and fever. | | symptom scores, cured rate, total effective rate |
| 28 | Liu ZP 2006^42^ | China | RCT | Shenmen (TF4), Internal Genitals (TF2), External Genitals (HX4), Liver (CO12), brain, and Kidney (CO10). | Group 1 (n=160, years: 13～25): Semen vaccariae seeds were sticked on the acupressure points by using adhesive plasters, and manually pressed 2 ～ 5 times a day. Those who feel pain again in the next menstrual cycle continue to be treated with this method. The course of treatment lasted for 3 menstrual cycles.  Group 2 (n=160, years: 13～26): Take Fenbid or indomethacin orally at the time of pain, 3 times a day, and intramuscularly inject pingtongxin if the pain does not abate for 4 to 6 hours. Those who feel pain again in the next menstrual cycle continue to be treated with this method. The course of treatment lasted for 3 menstrual cycles. | Press to local slight pain and fever. | | cured rate, total effective rate |
| 29 | Zhang L 2012^43^ | China | RCT | Shenmen (TF4), Internal Genitals (TF2), sympathesis (AH6a), Liver (CO12), subcortex (AT4), and Endocrine (CO18) | Group 1 (n=81, years: 14 ～ 33): treated with Fenbid (0.3 g/once, twice a day for 5 dyas). Meanwhile, semen vaccariae seeds were sticked on the acupressure points by using adhesive plasters, and manually pressed 2 ～ 4 minutes for a once, 4 ～ 5 times a day, 6 times each menstrual cycle. The course of treatment lasted for 3 menstrual cycles.  Group 2 (n=94, years: 14 ～ 33): treated with oral administration of Fenbid alone, and the dosage and course were the same as those of Group 1. | Press to swollen, numbness, or local slight pain and fever. | | cured rate, total effective rate |
| 30 | Yu F 2022^44^ | China | RCT | Shenmen (TF4), Internal Genitals (TF2), Kidney (CO10), Liver (CO12), subcortex (AT4), and Endocrine (CO19) | Group 1 (n=50, years: 21.75±1.83): treated with health education; meanwhile, semen vaccariae seeds were sticked on the acupressure points by using adhesive plasters, and manually pressed 1 time each menstrual cycle. The course of treatment lasted for 3 menstrual cycles.  Group 2 (n=50, years: 21.49±1.78): health education. | NR | | VAS, TCM symptom scores |
| 31 | Kong AJ 2016^45^ | China | RCT | Internal Genitals (TF2), Kidney (CO10), Liver (CO12), Endocrine (CO20), and abdomen (AH8). | Group 1 (n=50, years: 24.5±3.7): based on the treatment with Chinese herb decoction (taken orally, 1 dose per day, 3 times a day), Semen vaccariae seeds were sticked on the acupressure points by using adhesive plasters from one side to another every other day, and manually pressed 60 minutes for a once, 5 ～ 6 times a day, 7 days each menstrual cycle. The course of treatment lasted for 4 menstrual cycles.  Group 2 (n=100, years: 25.3±4.1): treated with Chinese herb decoction alone, and the dosage and course were the same as those of Group 1. | Press to swollen, or local slight pain and fever. | | cured rate, total effective rate |
| 32 | Wang SL 2016^46^ | China | RCT | Internal Genitals (TF2), sympathesis (AH6a), Endocrine (CO20), and shenmen (TF4). | Group 1 (n=30, years: 22.34±4.91): treated with acupoint application of Chinese herb [acupoints: Taichong (LR3), Guangyuan (RN4), Sanyinjiao (SP6), Xuehai (SP10), Hegu (LI4)], and auricular acupressure (Semen vaccariae seeds were sticked on the acupressure points by using adhesive plasters, and manually pressed 40 times for a once, 3 ～ 4 times a day, from 7 days before the beginning of the menstrual cycle to 7 days after menstruation for 3 menstrual cycles);  Group 2 (n=30, years: 22.34±4.91): treated with acupoint application of Chinese herb alone;  Group 3 (n=30, years: 22.34±4.91): treated with auricular acupressure alone. | Press to swollen, numbness, or local slight pain and fever. | | cured rate, total effective rate |
| 33 | Liu YD 2016^47^ | China | RCT | Internal Genitals (TF2), sympathesis (AH6a), Kidney (CO10), Liver (CO12), shenmen (TF4), Endocrine (CO20), and abdomen (AH8). | Group 1 (n=25, years: NR): treated with Chinese herb decoction [Luo's algomenorrhea prescription, 1 dose a day for 10 days from 7 days before the beginning of the menstrual cycle), and auricular acupressure (Semen vaccariae seeds were sticked on the acupressure points by using adhesive plasters, and manually pressed 30 ～ 60 seconds for a once, 3 ～ 5 times a day, from 7 days before the beginning to the of the menstrual cycleuntil the symptoms of dysmenorrhea were completely relieved, a total of 3 menstrual cycles);  Group 2 (n=25, years: NR): treated with Chinese herb decoction (Luo's algomenorrhea prescription) alone;  Group 3 (n=25, years: NR): treated with Chinese patent medicine (Tiaojing Huoxue Tablet, 5 tablets per once, 3 times a day for 10 days), The course of treatment lasted for 6 menstrual cycles. | Press to swollen, or local slight pain and fever. | | cured rate, total effective rate |
| 34 | Wang XX 2014^48^ | China | RCT | Internal Genitals (TF2), sympathesis (AH6a), Kidney (CO10), Liver (CO12), subcortex (AT4), Endocrine (CO20), and spleen (CO13). | Group 1 (n=60, years: 17.4±3.5): Semen vaccariae seeds were sticked on the acupressure points by using adhesive plasters from one side to another every 3 days, and manually pressed 3 ～ 5 seconds per an acoupoint for a once, 5 times a day, 6 days each menstrual cycle. The course of treatment lasted for 4 menstrual cycles.  Group 2 (n=50, years: 16.5±3.2): treated with Danggui Shaoyao decoction alone, and the dosage and course were the same as those of Group 1. | Press to swollen, numbness, or local slight pain. | | symptom scores, cured rate, total effective rate |
| 35 | Qiao L 2017^49^ | China | RCT | Shenmen (TF4), Internal Genitals (TF2), Kidney (CO10), Liver (CO12), subcortex (AT4), and Endocrine (CO20). | Group 1 (n=20, years: 16～30): treated with acupuncture; acupoints: Guanyuan (RN4), Diji (SP8), and Sanyinjiao (SP6), 30 minutes/ once, once a day for 7 days, from 5 days before the beginning to the second day of the menstrual cycle. The course of treatment lasted for 3 menstrual cycles.  Group 2 (n=20, years: 16～30): treated with wrist-ankle acupuncture, 30 minutes/ once, once a day for 7 days, from 5 days before the beginning to the second day of the menstrual cycle. The course of treatment lasted for 3 menstrual cycles.  Group 3 (n=20, years: 16～30): treated with auricular acupressure, (Semen vaccariae seeds were sticked on the acupressure points by using adhesive plasters, and manually pressed 5 minutes every 3 hours from 5 days before the beginning to the second day of the menstrual cycle. The course of treatment lasted for 3 menstrual cycles).  Group 4 (n=20, years: 16～30): treated with Chinese herb decoction (Luo's algomenorrhea prescription) alone;  Group 5 (n=40, years: 16～30): treated with indomethacin tablets, taken orally, 25 mg for a once, twice a day from the onset of menstrual pain until the symptoms of dysmenorrhea were completely relieved, a total of 3 menstrual cycles. | NR | | symptom scores, cured rate, total effective rate |

RCT: randomized controlled trial; NR: not reported; VAS: visual analogue scale; MDQs: Short-form Menstrual Distress Questionnaire; NO: nitric oxide.

**Supplementary eTable 2 Sensitivity analysis for time to first flatus.**

| **Outcomes** | **Number of studies** | **Begg's test** | **Egger's test** |
| --- | --- | --- | --- |
| Cured rate | 26 | 0.831 | 0.9077 |
| Total effective rate | 28 | 0.018 | 0.018 |
| VAS | 10 | 1.00 | 0.850 |
| MDQs | 2 | 1 | Not applicable |
| symptom scores | 10 | 0.371 | 0.225 |
| Serum NO level | 2 | 1 | Not applicable |

VAS: visual analogue scale; MDQs: Short-form Menstrual Distress Questionnaire; NO: nitric oxide.
